# Supplementary figures and images for: When predict can also explain: Few-shot prediction to select better neural latents
Source: PLoS Comput Biol. 2025 Dec 30;21(12):e1013789. doi: 10.1371/journal.pcbi.1013789 (PMC12779162; doi:10.1371/journal.pcbi.1013789)

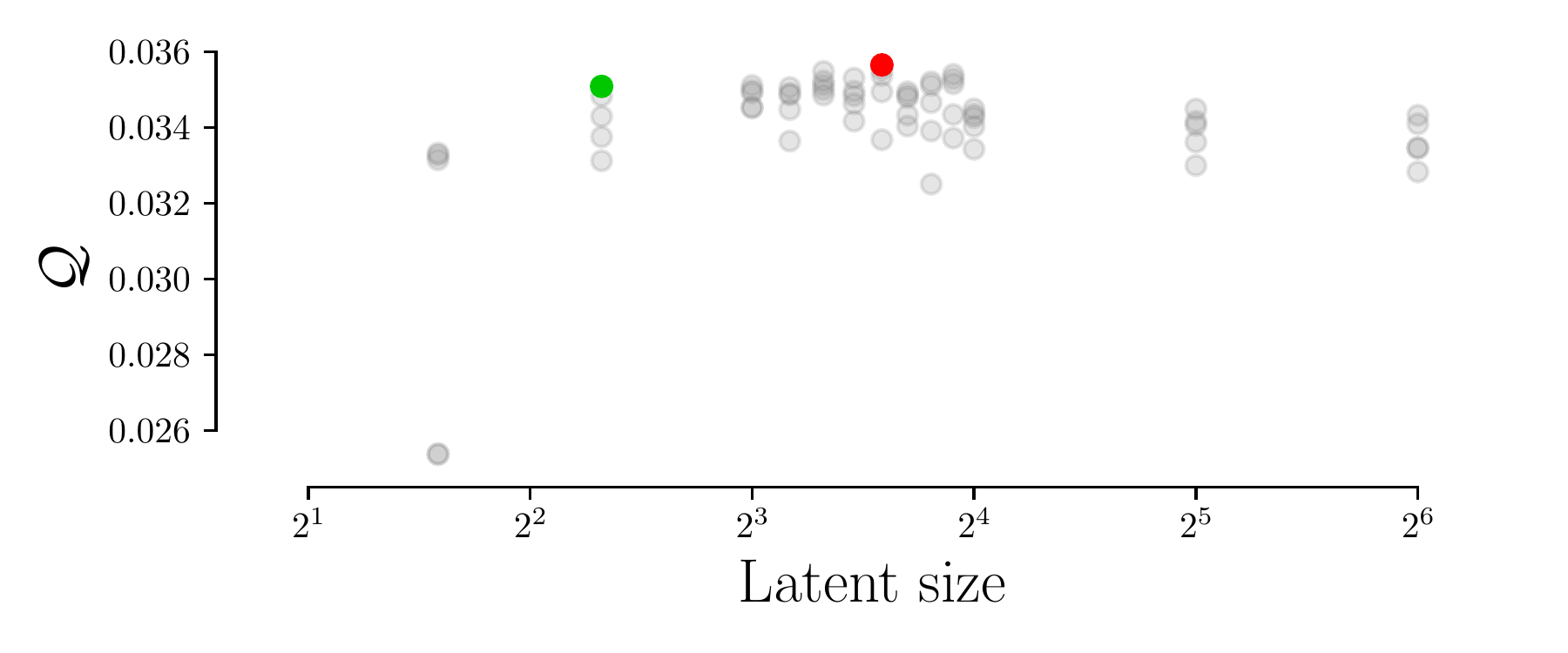

Supplement: S1 Fig — Finding the correct model is not just about tuning the latent size hyperparameter. NODE SAE students over a range of sizes (5-15) achieve high co-smoothing on the same 64-unit noisy GRU performing 3BFF teacher (Methods). (TIFF) [file pcbi.1013789.s001.tif]

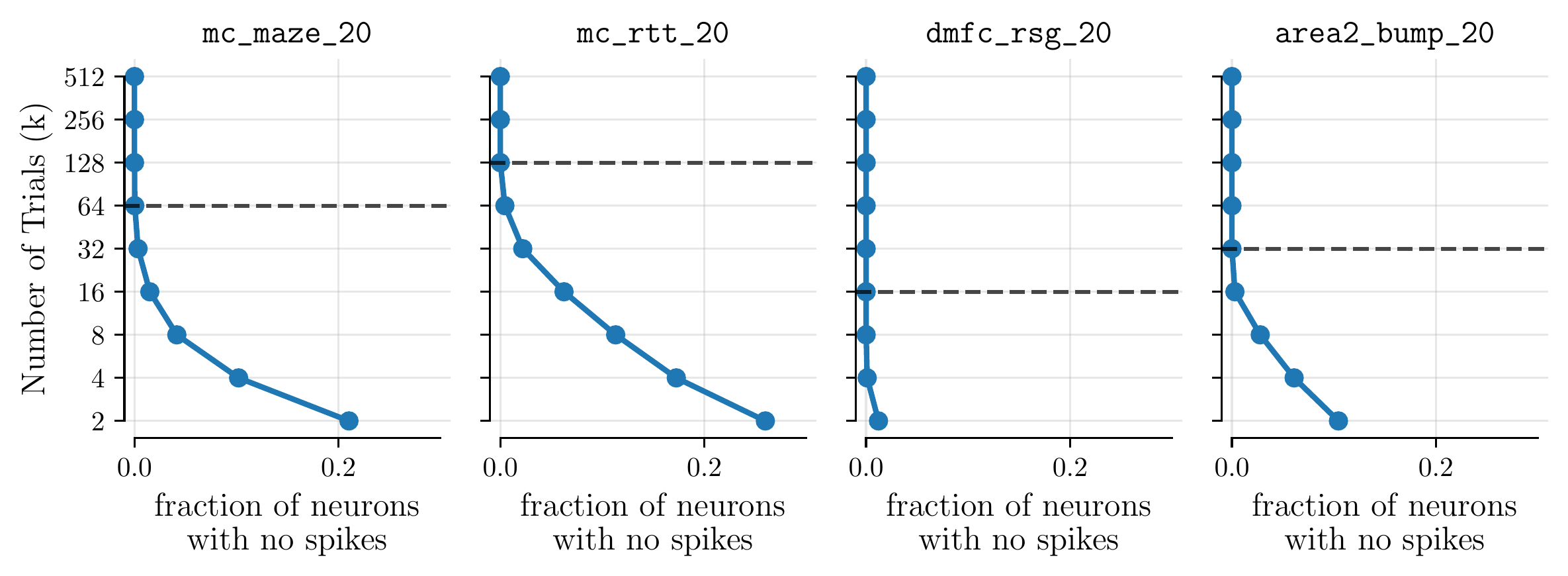

Supplement: S2 Fig — Our theoretical analysis in “Why does few-shot work?” reveals that extraneous models are best discriminated when the shot number, k, is small. So how small can we go? In the case of sparse data like neural spike counts we may obtain k-trial subsets in which some neurons are silent. In this scenario the few-shot decoder g′ receives no signal for those neurons. To avoid this pathological scenario, for each dataset, we pick the smallest possible k that ensures that the probability of encountering silent neurons in a k-trial subset is safely near zero. This must be computed for each dataset independently since some datasets are more sparse than others. We compute the frequency of such silences for different k, for each NLB [6] dataset, and show the values of k (dashed lines) chosen for the analysis in the main text. (TIFF) [file pcbi.1013789.s002.tif]

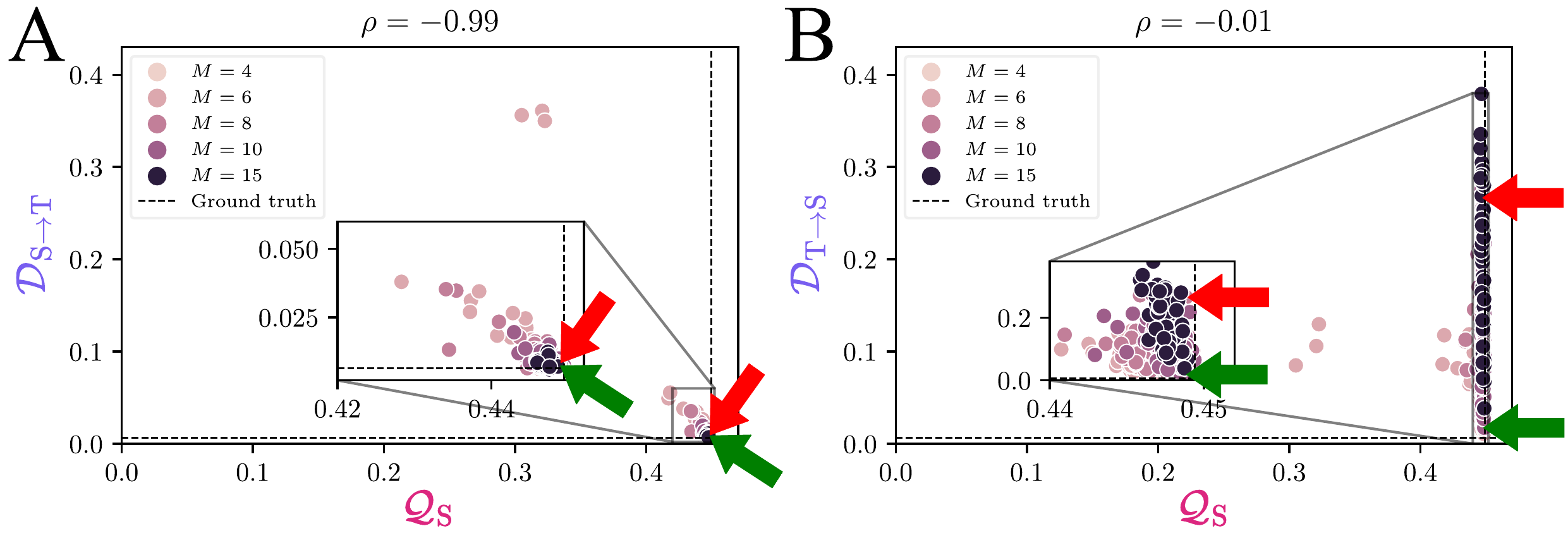

Supplement: S3 Fig — In the main text, we show how good prediction of held-out neural activity, i.e., co-smoothing, does not guarantee a match between model and true latents. We did this in the student-teacher setting of RNNs and NODE SAEs (Fig 2). Here we replicate the results in HMMs (see Methods). Similar to Fig 2, several students HMMs are trained on a dataset generated by a single teacher HMM, a noisy 4-cycle. The Student→Teacher decoding error DS→T is low and tightly related to the co-smoothing score. The Teacher→Student decoding error DT→S is more varied and uncorrelated to co-smoothing. The arrows mark the “Good” and “Bad” transition matrices shown in the Fig 2 (lower). (TIFF) [file pcbi.1013789.s004.tif]

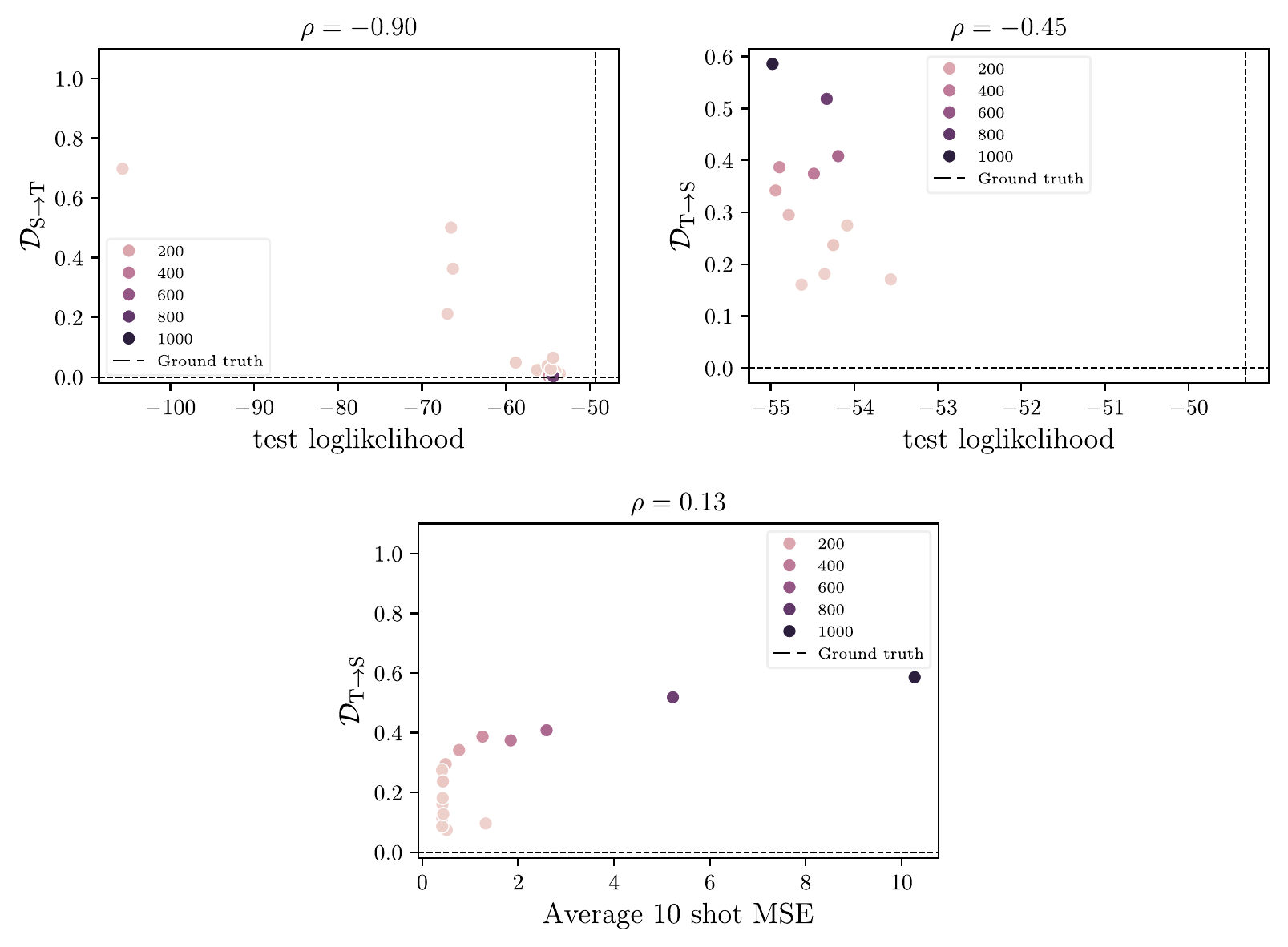

Supplement: S4 Fig — We demonstrate that our results are not unique to the RNN or HMM settings by simulating another simple scenario: linear gaussian state space models (LGSSM), i.e., Kalman Smoothing. The model is defined by parameters (μ0,Σ0,F,G,H,R). A major difference to HMMs is that the latent states z∈RM are continuous. They follow the dynamics given by:z0~N(μ0,Σ0) (54)zt~N(Fzt−1+b,G) (55)xt~N(Hzt+c,R) (56) Given these dynamics, the latents z can be inferred from observations x using Kalman smoothing, analogous to (15). Here we use the jax based dynamax implementation. We use a teacher LGSSM with M = 4, with parameters chosen randomly (using the dynamax defaults) and then fixed. Student LGSSMs are also initialised randomly and optimised with Adam [51] to minimise negative loglikelihood on the training data (see the dataset dimensions section for dimensions of the synthetic data set). DS→T and DT→S is computed with linear regression (sklearn.linear_model.LinearRegression) and predictions are evaluated against the target using R2 (sklearn.metrics.r2_score). We define Du→v:=1−(R2)u→v. Few-shot regression from z to xk--out is also performed using linear regression. In line with our results with RNNs and HMMs (Fig 2 and Fig 4), we show that among the models with high test loglikelihood (>–55), DS→T, but not DT→S, is highly correlated to test loglikelihood, while DT→S shows a close relationship to Average 10 shot MSE error. For these Linear Gaussian State Space Models, we report loglikelihood instead of co-smoothing, and k-shot MSE instead of k-shot co-smoothing, demonstrating the same pattern of results across different model classes. (TIFF) [file pcbi.1013789.s005.tif]

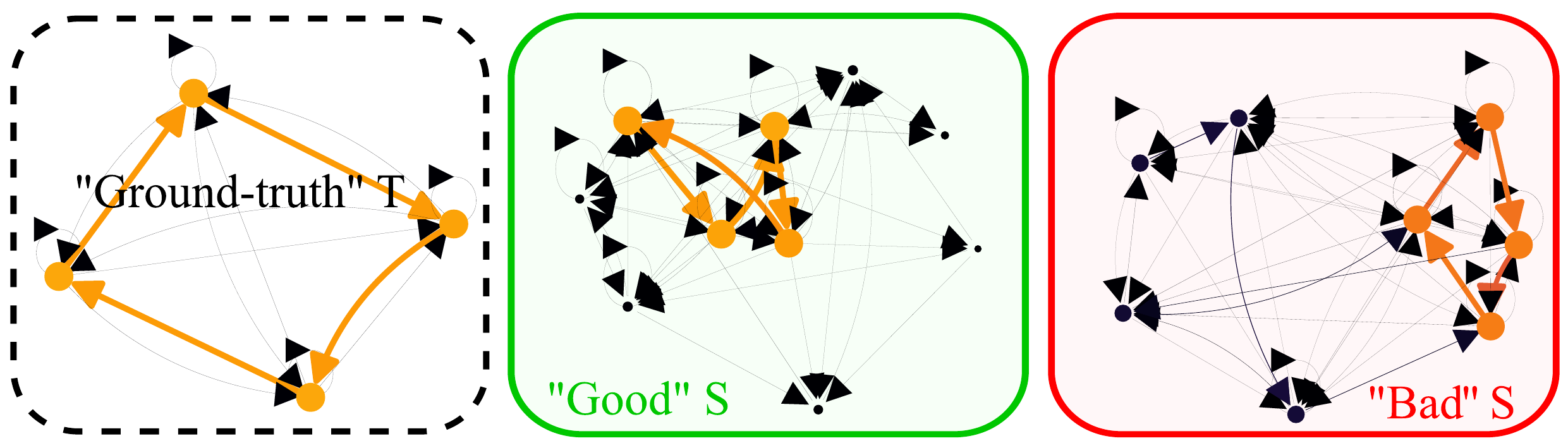

Supplement: S5 Fig — l In the main text Fig 2 we visualised the teacher and two student HMMs as graphs of fractional traffic volume on states and transitions. For clarity we dropped the low probability edges with values lower than 0.01. We also show the same models with all the edges visualised, including the low probability transitions that were omitted in the main text figure for clarity. (TIFF) [file pcbi.1013789.s006.tif]

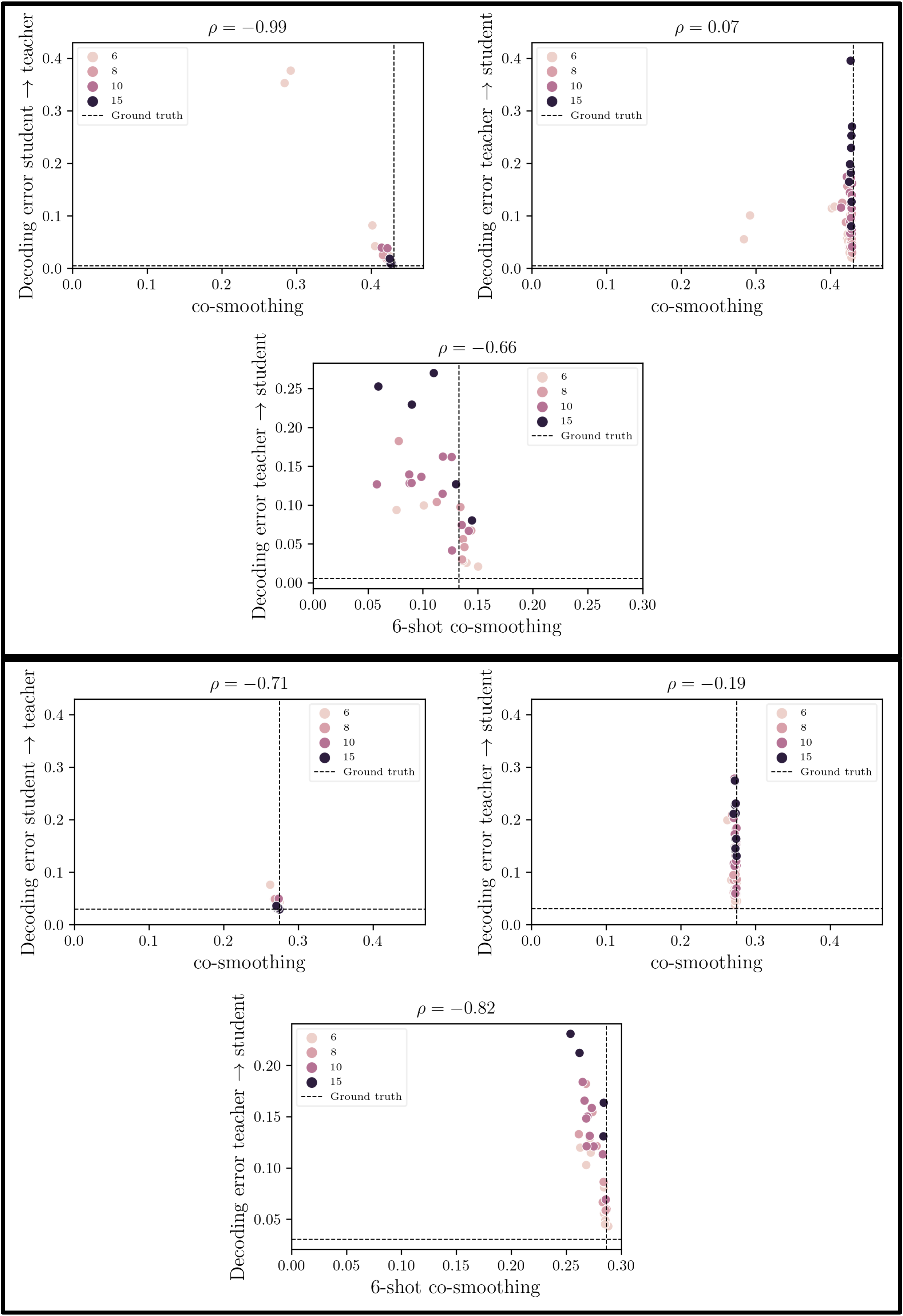

Supplement: S6 Fig — Few-shot co-smoothing is a more difficult metric than standard co-smoothing. Thus, it might seem that any increase in the difficulty of will yield similar results. To show this is not the case, we use standard co-smoothing with fewer held-in neurons. The score is lower (because it’s more difficult), but does not discriminate models. We demonstrate this through two variations of HMM student-teacher experiments. In the first variation, we increase the number of held out neurons from Nout=50 to Nout=100, making the co-smoothing problem harder. The top three panels show: (1) decoder student-teacher original simple, (2) decoder teacher-student original simple (same as main text Fig 1CD), and (3) decoder teacher-student 6-shot best (same as main text Fig 4B). In the second variation, we decrease the number of held-in and held-out neurons to Nin=5, Nout=5, Nk−out = 50, further increasing difficulty. The bottom three panels show the same three decoder configurations as the top row. While the score does decrease because the problem is harder, co-smoothing is still not indicative of good models while few-shot co-smoothing remains discriminative. (TIFF) [file pcbi.1013789.s007.tif]

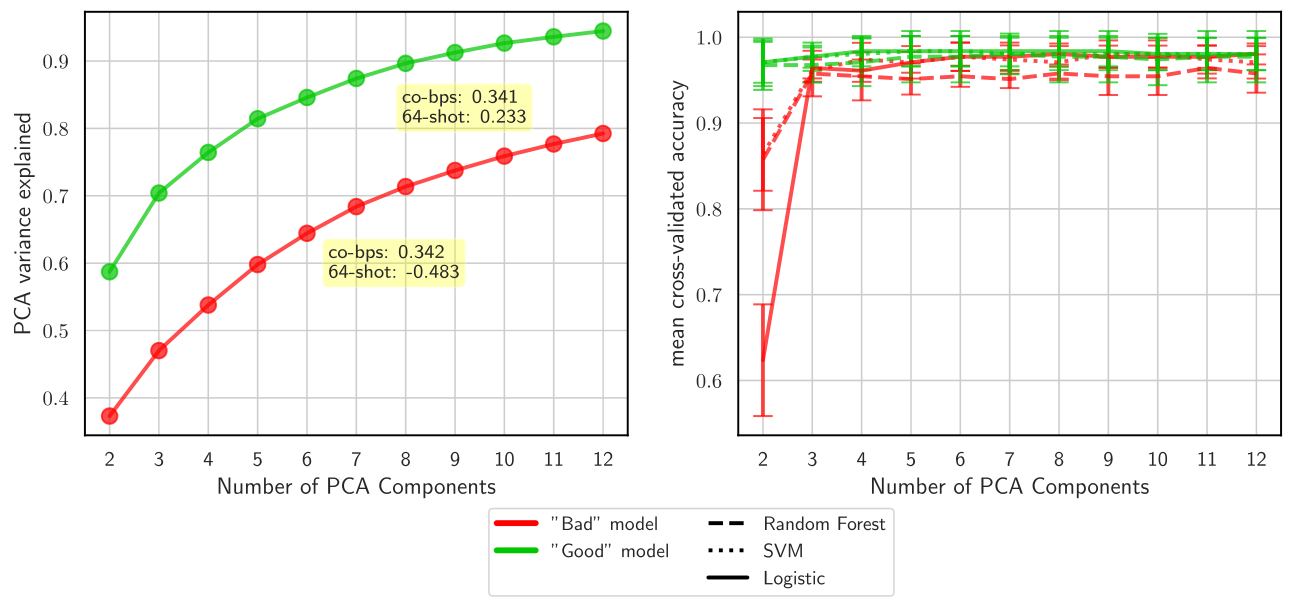

Supplement: S7 Fig — In main text Fig 7(lower panel), we compare two STNDT models trained on mc_maze_20 that perform identically under standard co-smoothing but diverge under 64-shot co-smoothing. Projecting their latents onto the top two principal components reveals differences in trajectory smoothness and task-condition separation. Quantitatively, the “Bad” model exhibits higher latent dimensionality, as reflected by the slower growth of variance explained across PCs (left panel), and yields poorer binary classification of maze barrier presence—especially when using only the top two principal components (right panel). (TIFF) [file pcbi.1013789.s009.tiff]
