# Supplementary material for: When predict can also explain: Few-shot prediction to select better neural latents
Source: PLoS Comput Biol. 2025 Dec 30;21(12):e1013789. doi: 10.1371/journal.pcbi.1013789 (PMC12779162; doi:10.1371/journal.pcbi.1013789)
Supplement: S1 Appendix — (PDF) [file pcbi.1013789.s003.pdf]

S1 Appendix.

Decoding across HMM latents: fitting and evaluation. Consider two HMMs u and v, of sizes $M(u)$ and $M(v)$, both candidate models of a dataset $\mathcal{X}$. Following (**??**), each HMM can be used to infer latents from the data, defining encoder mappings f^u and f^v. These map a single trial i of the data $(_{:,\mathrm{in}})^{(i)} \in \mathcal{X}$ to $(\boldsymbol{\xi}_t^{(i)})_u$ and $(\boldsymbol{\xi}_t^{(i)})_v$.

We now perform a multinomial regression from $(\boldsymbol{\xi}_t^{(i)})_u$ to $(\boldsymbol{\xi}_t^{(i)})_v$.

$$\boldsymbol{p}_t^{(i)} = h\left(\left(\boldsymbol{\xi}_t^{(i)}\right)_u\right) \tag{1}$$

$$h(\xi) = \sigma(W\boldsymbol{\xi} + \boldsymbol{b}) \tag{2}$$

where $W \in \mathbb{R}^{M(v)\times M(u)}$, $\boldsymbol{b} \in \mathbb{R}^{M(v)}$ and σ is the softmax. During training we sample states from the target PMFs $(z_t^{(i)})_v \sim (\boldsymbol{\xi}_t^{(i)})_v$ thus arriving at a more well know problem scenario: classification of $M(v)$-classes. We optimize W and $\boldsymbol{b}$ to minimise a cross-entropy loss to the target $(\hat{z}_t^{(i)})_v$ using the `fit()` method of `sklearn.linear_model.LogisticRegression`.

We define decoding error, as the average Kullback-Leibler divergence D_{KL} between target and predicted distributions:

$$\mathcal{D}_{u\to v} := \frac{1}{S^{\mathrm{test}}T} \sum_{i\in\mathrm{test}} \sum_{t=1}^{T} D_{KL}\left(\boldsymbol{p}_t^{(i)}, (\boldsymbol{\xi}_t^{(i)})_v\right) \tag{3}$$

where D_{KL} is implemented with `scipy.special.rel_entr`.

In S3 Fig. the data X is sampled from a single teacher HMM, T, and we evaluate $\mathcal{D}_{\mathrm{T}\to\mathrm{S}}$ and $\mathcal{D}_{\mathrm{S}\to\mathrm{T}}$ for each student notated simply as S.
