# Supplementary material for: When predict can also explain: Few-shot prediction to select better neural latents
Source: PLoS Comput Biol. 2025 Dec 30;21(12):e1013789. doi: 10.1371/journal.pcbi.1013789 (PMC12779162; doi:10.1371/journal.pcbi.1013789)
Supplement: S2 Appendix — (PDF) [file pcbi.1013789.s008.pdf]

S2 Appendix.

Time cost of computing few-shot co-smoothing

The compute time depends on several factors. It scales with the number of trials k, T the number of samples per trial, the number of neurons $N^{k\text{-out}}$ and number of repeated resamples s and fitting of the regressor. Each repetition and neuron is an independent regression and therefore can computed in parallel, provided the compute resources are available. For `mc_maze_20` each repetition for $k = 64$, took 0.62 ± 0.06 seconds and we iterated over $s = 12$ such regressions, requiring a total of 7.44 seconds.
