# Supplementary material for: When predict can also explain: Few-shot prediction to select better neural latents
Source: PLoS Comput Biol. 2025 Dec 30;21(12):e1013789. doi: 10.1371/journal.pcbi.1013789 (PMC12779162; doi:10.1371/journal.pcbi.1013789)
Supplement: S3 Appendix — (PDF) [file pcbi.1013789.s010.pdf]

S3 Appendix.

Illustrative example of the difference between cycle consistency and cross-decoding

In the main text, to validate our few-shot co-smoothing on real data, we used two alternative proxies to evaluate model extraneousness when the ground truth $\mathcal{D}_{\mathrm{T}\to\mathrm{S}}$ is not available. The first is cycle consistency, $\mathcal{D}_{\boldsymbol{r}\to\hat{\boldsymbol{z}}}$, and the second is the cross-decoding column average, $\langle\mathcal{D}_{u\to v}\rangle_u$. In general, the two measures were highly correlated, but there were systematic differences in some cases. This implies that there could be scenarios in which one measure is superior to the other. Here we illustrate a simple circumstance in which only cross-decoding identifies model extraneousness. Consider a setting where the true latent, $\boldsymbol{z}$, is constant in time, e.g., a fixed point. Consider two candidate models, one in which the inferred latents $\hat{\boldsymbol{z}}$ and predicted rates $\boldsymbol{r}$ oscillate, and one in which latents and rates are constant, but the rates are at a suboptimal value such that both models have similar co-smoothing. Such a suboptimal value can arise due to noise or interference from other trials. Clearly, the first latent is extraneous relative to the second, but cycle-consistency, $\mathcal{D}_{\boldsymbol{r}\to\hat{\boldsymbol{z}}}$, would fail to identify it as both model latents are decodable from their respective rate predictions. In this scenario, cross-decoding would differentiate the two: due to the difference in $\mathcal{D}_{1\to 2}$ and $\mathcal{D}_{2\to 1}$.
