# Supplementary material for: When predict can also explain: Few-shot prediction to select better neural latents
Source: PLoS Comput Biol. 2025 Dec 30;21(12):e1013789. doi: 10.1371/journal.pcbi.1013789 (PMC12779162; doi:10.1371/journal.pcbi.1013789)
Supplement: Attachment — Submitted filename: Few shot rebuttal.pdf [file pcbi.1013789.s012.pdf]

Rebuttal 2: “When predict can also explain: few-shot prediction to select better neural latents”

We thank the reviewers for their valuable feedback. Here we summarise the changes and refinements to the manuscript.

Reviewers' comments:

Reviewer's Responses to Questions

Reviewer #2: The revisions address my issues with the first submission, and I approve of the additional changes.

Minor comments:
- In the section "Why does few-shot work?", you present the linear regression case first without saying that's what you're doing. That is, on p7 line 142, you introduce three models (LR, HMM, prototype). The next paragraph would be clearer if you started it with "For the linear regression case..." or similar.

Thanks, we added this.
- In Figure 5, it took me a second to notice that in Panel A higher is better (likelihood), while in Panels B and C lower is better (error). So I didn't get right away that all three were showing the same trend with respect to extraneous noise. It would be helpful just to state it explicitly in the text and/or caption.

Now added to the figure.
- On p8 lines 156-158, you compare the methods in terms of their bias/variance decompositions. I assume you are referring to the analysis in the Methods sections "Theoretical analyis of...". If so, please refer the reader to these methods section (again).

We now added links to specific equations.

Reviewer #3: This manuscript highlights the problem of extraneous/spurious dynamics in latent variable models and introduces two evaluation approaches for identifying it: few-shot co-smoothing and cross-decoding. This problem is important and hampers interpretation and scientific conclusions drawn from these models, so suitable evaluation frameworks are a timely and valuable contribution. After the first revision, the manuscript is much clearer and more well-motivated. However, I do still have a few questions/comments about the work:

1. Given that 1) few-shot co-smoothing is still used in conjunction with standard co-smoothing, and 2) cycle consistency and cross-decoding also indicate presence of spurious dynamics (but not co-smoothing quality), what does few-shot co-smoothing exactly offer that the combination of co-smoothing and e.g., cycle consistency does not?

As detailed in the response below, few-shot co-smoothing is a prediction-based measure, whereas cycle consistency and cross decoding require access to the latents.

Can few-shot co-smoothing be sufficient alone, without also evaluating standard co-smoothing?

No. In the student-teacher setting we showed that high co-smoothing is strongly linked to D_{S->T}, but not D_{T->S}, ensuring that student captures that teacher latents (Fig 2 top-left) but not vice-versa. We then showed that few-shot co-smoothing is linked to D_{T->S}, when standard co-smoothing is kept fixed. For the best match between teacher and student, we desire both D_{T->S} and D_{S->T}. This requires evaluating both standard co-smoothing and few-shot co-smoothing. This is mentioned in "Results: Few-shot prediction selects better models".

Why is it important to specifically have a prediction-based metric for parsimony of latents?

Prediction on held-out data is the lingua franca of modern machine learning benchmarks. The Neural Latents Benchmark [Pei et al., 2021] makes this explicit via the EvalAI platform, where participants upload predictions that are evaluated against a private test set. and few-shot prediction has recently been adopted for iBCI benchmarking [Karpowicz et al., 2024], where generalization to new settings with limited data is crucial. Few-shot generalization is similarly important in other domains (e.g., language; Brown et al., 2020). By grounding parsimony in predictive performance, few-shot co-smoothing integrates naturally with existing benchmarking frameworks and is agnostic to model architecture.

2. It is stated in the text that cycle consistency relies on the models having "perfect co-smoothing" or having "rate predictions [that are] perfect proxies of the true dynamics," while cross-decoding does not. However, it is also stated in the text that cross-decoding also relies on the assumption that "high co-smoothing models contain the teacher latent." How is this assumption different from that of cycle consistency?

This is indeed a subtle but important distinction. **Cycle consistency** involves mapping a model's *own rate predictions* back to its latents, effectively treating those rate predictions as a *proxy* for the ground truth. This is only fair to all models if their rate predictions are near identical—i.e., if they have achieved near perfect high co-smoothing. Otherwise, the rate predictions can differ, leaving room for extraneous dynamics to persist in some models. In such cases, cycle-consistency would fail to discredit those extraneous dynamics.

Cross-decoding, by contrast, provides a fairer comparison across models. All models have a *common jury*: the latents of the entire set of trained models**.** In this regard, it does not require perfect co-smoothing.

We added an appendix (S3) that discusses this point.

3. Though linear-exponential-poisson readouts are most common, some LVMs do not have this emissions model (e.g., linear-softplus, MLP). I assume this makes (linear) cross-decoding not directly applicable, and I wonder if few-shot co-smoothing scores are comparable across readout models. I assume the few-shot generalization behavior would vary, especially for a higher parameter count, neural network-based readout as in ODIN (Versteeg et al. 2023), so I would appreciate some empirical exploration and/or discussion of this limitation (if I am correct in assuming that it is a limitation).

Indeed, we only studied linear–exponential–Poisson readouts for the real neural data. However, our theoretical results span three different emission settings (linear regression, Bernoulli HMM, and prototype classification; Fig. 5) and suggest that the phenomenon itself is quite general: models with extraneous dynamics are less data-efficient, regardless of the specific emission model. When comparing architectures with different emission models, each would in principle require its own few-shot learning

(FSL) procedure. The resulting few-shot co-smoothing score then reflects not only the quality of the latent representation but also the expressiveness of the emission model and the efficiency of the FSL algorithm.

Although in this work we focus on *linear cross-decoding*, the method could be extended to more expressive mappings if desired, since cross-decoding does not restrict the amount of training data.

For LVMs with deep MLP decoders (e.g., ODIN; Versteeg et al., 2023), we agree that few-shot generalization may degrade due to the more complex mapping from latents to neural data. This may be viewed as a limitation of our approach (we added a sentence in discussion), but we note two mitigating points:

1. In these cases, one can instead treat the *last layer activations* of the MLP as the effective latents, so that the decoder is again a single linear layer.
2. Alternatively, one might argue that a simple mapping to neural activity is itself a desirable property of a good latent representation – e.g., allowing better decoding – so poor few-shot scores in these settings could be informative rather than merely limiting.

4. Though it is maybe obvious, I think the two-bit flip flop is potentially a good example to briefly discuss the consequence of spurious dynamics on accurate interpretation of the system. Are the red and green stars in Fig 2 unstable and stable fixed points? Do the spurious dynamics in the "bad" model lead to incorrect fixed point topology (as computed in Maheswaranathan et al. 2019, for example)?

Red and green stars are indeed unstable and stable fixed points, thanks. We now annotated the figure.

Yes, the "bad" model indeed has incorrect fixed point topology due to the absence of any fixed points.

5. Though maybe only directly applicable to models with strictly linear emissions models, I would appreciate some discussion of Procrustes-style metrics from Alex Williams and others, which also penalize spurious dynamics without needing two separate metrics.

Indeed Procrustes-style metrics may resolve the need for two separate metrics as they are symmetric. However, the original procrustes-style shape metrics (Williams et al Neurips 2021) are not ideal for single latents inferred on single trials as they are formulated for deterministic data, e.g., after trial-averaging. This framework has recently been extended to stochastic representations assuming gaussian noise (Duong et al 2022). Co-smoothing is formulated with a Poisson firing rate model of the spike counts for neural spiking data.

6. I appreciate the qualitative difference in smoothness and separation of latents in Fig 7. Can you perform any quantitative evaluations that support this point? For example, can conditions be more accurately classified from initial conditions of the "good" model?

We now quantify this. We train a binary classier to classify the if the mc_maze trial was one conducted with or without barriers from the initial latent in each trial. We show that the "good" model is lower dimensional and achieves higher cross-validated accuracy, than the "bad" model, when using only the first two principal components of the latents. When we use a larger number of principal components, both models achieve similar accuracy. These results strengthen our claim that both models contain the ground-truth but the 'bad' model has extraneous dynamics, distributing the informative features into other PCs.

S7 Fig. now shows this.

7. Minor typo: disucssion (line 214)

Thanks, this is now fixed.

Have the authors made all data and (if applicable) computational code underlying the findings in their manuscript fully available?
The PLOS Data policy requires authors to make all data and code underlying the findings described in their manuscript fully available without restriction, with rare exception (please refer to the Data Availability Statement in the manuscript PDF file). The data and code should be provided as part of the manuscript or its supporting information, or deposited to a public repository. For example, in addition to summary statistics, the data points behind means, medians and variance measures should be available. If there are restrictions on publicly sharing data or code —e.g. participant privacy or use of data from a third party—those must be specified.

Reviewer #2: Yes

Reviewer #3: Yes

PLOS authors have the option to publish the peer review history of their article (what does this mean?). If published, this will include your full peer review and any attached files.

If you choose “no”, your identity will remain anonymous but your review may still be made public.

Do you want your identity to be public for this peer review? For information about this choice, including consent withdrawal, please see our Privacy Policy.

Reviewer #2: **Yes:** Emily P Stephen

Reviewer #3: No

[NOTE: If reviewer comments were submitted as an attachment file, they will be attached to this email and accessible via the submission site. Please log into your account, locate the manuscript record, and check for the action link "View Attachments". If this link does not appear, there are no attachment files.]

Figure resubmission:

While revising your submission, we strongly recommend that you use PLOS’s NAAS tool (https://ngplosjournals.pagemajik.ai/artanalysis) to test your figure files. NAAS can convert your figure files to the TIFF file type and meet basic requirements (such as print size, resolution), or provide you with a report on issues that do not meet our requirements and that NAAS cannot fix.

After uploading your figures to PLOS’s NAAS tool - https://ngplosjournals.pagemajik.ai/artanalysis, NAAS will process the files provided and display the results in the "Uploaded Files" section of the page as the

processing is complete. If the uploaded figures meet our requirements (or NAAS is able to fix the files to meet our requirements), the figure will be marked as "fixed" above. If NAAS is unable to fix the files, a red "failed" label will appear above. When NAAS has confirmed that the figure files meet our requirements, please download the file via the download option, and include these NAAS processed figure files when submitting your revised manuscript.

Reproducibility:

To enhance the reproducibility of your results, we recommend that authors of applicable studies deposit laboratory protocols in protocols.io, where a protocol can be assigned its own identifier (DOI) such that it can be cited independently in the future. Additionally, PLOS ONE offers an option to publish peer-reviewed clinical study protocols. Read more information on sharing protocols at https://plos.org/protocols?utm_medium=editorial-email&utm_source=authorletters&utm_campaign=protocols

Bibliography:

Pei, Felix, et al. "Neural latents benchmark'21: evaluating latent variable models of neural population activity." *arXiv preprint arXiv:2109.04463* (2021).

Karpowicz, Brianna M., et al. "Few-shot algorithms for consistent neural decoding (falcon) benchmark." *Advances in Neural Information Processing Systems* 37 (2024): 76578-76615.

Brown, Tom, et al. "Language models are few-shot learners." *Advances in neural information processing systems* 33 (2020): 1877-1901.

Williams, Alex H., et al. "Generalized shape metrics on neural representations." *Advances in neural information processing systems* 34 (2021): 4738-4750

Duong, Lyndon R., et al. "Representational dissimilarity metric spaces for stochastic neural networks." *arXiv preprint arXiv:2211.11665* (2022).
