# Supplementary material for: When predict can also explain: Few-shot prediction to select better neural latents
Source: PLoS Comput Biol. 2025 Dec 30;21(12):e1013789. doi: 10.1371/journal.pcbi.1013789 (PMC12779162; doi:10.1371/journal.pcbi.1013789)
Supplement: Attachment — Submitted filename: few shot rebuttal.docx [file pcbi.1013789.s011.docx]

# Rebuttal: “When predict can also explain: few-shot prediction to select better neural latents”

We thank the reviewers for taking the time and giving us valuable feedback. We made many changes and refinements to the manuscript, and believe it is much better due to this process.

Major changes are:

1. Using trained RNNs as the main ground truth example.
2. Adding three new experimental datasets to the analysis.
3. Improved graphics and text.

Below are detailed responses to the specific comments.

Reviewer's Responses to Questions

Reviewer #1: This manuscript introduces a novel and interesting few-shot prediction metric to enhance the alignment between inferred latent variables and the true latent. Such a tool holds significant potential for modern neuroscience research, particularly in the context of large-scale population recordings. Nevertheless, the paper lacks sufficient results to support their conclusion and the presentation of the work is kind of messy.

We thank the reviewer for appreciating the potential of our work, and for pointing us in directions we believe strengthened the results and the presentation.

1. When the authors challenge the common assumption on the effectiveness of co-smoothing, they can provide concrete examples in real neural data, instead of using a simplified HMM as a toy example.

We now use task-trained RNNs, along with four different real neural datasets.

2. line 95-96, "It is common to assume that being able to predict held-out parts of X will guarantee that the inferred latent aligns with the true one"

I don't think this is a common assumption. Although co-smoothing is commonly used for benchmarking LVMs, their purpose is to demonstrate their inferred latents recover the true latent signals in some way (e.g., rotation, permutation, linear combinations etc.).

Actually, this is consistent with your statment in line 101 "we hypothesize that good prediction guarantees that the true latents are contained within the inferred ones".

We agree that the purpose is to show “latents recover the true latent in some way”. While rotations and permutations are indeed common transformations to the latents, our reading of the literature is that discarding extraneous dynamics from the inferred latents are not commonly part of this allowed set.

3. Over-parameterization usually achieves better prediction scores. By regularizing the model to have pasimonious parameters, we may achieve good alignment between the inferred latents and the true latents. This can be verified in Fig 1E, small number of latent yields smaller D_{T->S} values. I think the authors should also comment on their few-shot approach vs model selection approach (using AIC/BIC or other regularizers described in line 18-20). One is using limited data, while the other is using limited model parameters.

Indeed, some of the models with few latents in the previous Fig 1E also had small D_{T->S} values. But there were also models with more parameters and similar or better scores. This is similar in the new RNN results. In real data, we find that co-smoothing keeps improving with more parameters, which is consistent with the modern use of overparametrized networks.

Regarding model selection, the aim of our work was to provide a tool that only uses prediction metrics and is not allowed to peek inside the model and count parameters. This is also mentioned in the paragraph in lines 21-24.

4. The organization of the manuscript needs major revision. The manuscript put too much space to discuss the toy example HMM and fail to validate its applicability in real LVM on neural data, whose latent are continuous values, instead of discrete states. Most people will agree 'high prediction score not necessarily yields good alignment with the true latents', thus there is no need to use 1 page to verify this with an HMM model. Simply using a LFADS or STNDT results to show the diversity of inferred latent dynamics suffices. Or you can use simulated data to provide ground truth latent.

We thank you for the suggestion on simulated data, and now use task trained RNN exactly for this purpose. The synthetic example makes things cleaner and easier to understand, and therefore we prefer to begin with it. We still briefly present HMMs, because it’s easier to see extraneous latent states in this setting.

According to the suggestion, we now put more emphasis on real data (four datasets), and on continuous models (why few shot works section and the RNN example).

5. In the discussion of 'why does few-shot work', you can just describe the more insightful comments. The current version is too lengthy and obscure. You can also move them to supplementary materials. I think the manuscript should include more examples of neural data to validate the performance of this few-shot prediction score.

This section was now shortened to just show construction and results, and also we moved the continuous version to the main text and the discrete HMM theory to supplement.

As we mentioned above, we did add more examples of neural data.

6. The few-shot approach is very similar to bagging in ensemble learning, with an aim of reducing variance of the model estimation. In bagging, a random sample of data in a training set is selected with replacement. After generating several data samples, these weak models (g' in your work) are then trained independently. Proably you can comment your methods by connecting it with bagging.

There is indeed some resemblance, but the aim is different. Bagging is a method to improve the prediction quality of weak models by aggregating them. We take a strong model and make it weaker on purpose to reveal extraneous dynamics. Furthermore, the way in which we make it weak (small training data) is crucial, because making the prediction problem harder in other forms does not reveal these extraneous dynamics (see S7 Appendix)

7. The few-shot prediction approach, which is the main contribution of the manuscript, is not clearly described.
We now include a more clear description in the Methods section.
a. Line 152, you used N^{k-out}. What's the typical value for N^{in}, N^{out}, N^{k_out}?
These values are provided in S8 appendix.
b. The caption of Fig 3B says that 'f and g are frozen'. If so, how does Q^k help with the model training? Are you only using few-shot prediction for model evaluation?
Yes – only for evaluation.
b. If the few-shot prediction is simply used for evaluation, the metric is only used for comparing the inferred models. What if all models fail to align with the true latent?
As with most benchmarks, we can compare models to each other using a common metric. The standard co-smoothing metric selects models that are expressive enough to fit the data. Our few-shot metric can take these models and further rank them according to their similarity to the true latent, even without access to this ground truth. We expect all models to fail aligning **completely** with the true latent, but can select the best option.

8. The format of the manuscripts needs huge changes. It looks like a rash change from conference submission. The authors should follow the PLOS journal format and organize their manuscript according to journal standards. For example, the supporting information has subsections with a prefix of A-Z, and different sections are like randomly compiled contents without inherent connections.

We changed all the places that deviate from PLoS format.

9. The time cost in computing few-shot prediction metric should be presented or discussed.

We now report the computational time cost of the few-shot evaluation in S10 Appendix.

10. What if the data has no trial structure? For example, LFADS uses single trial to infer the latents. Maybe you have different definitions of trials?
This is an important point. First, note that the RTT datasets does not have a trial structure. It consists of a long sequence of saccades, and is broken into “trials” by simply dividing it to arbitrary segments with the same duration.

Second, the few-shot aspect of the work is blind to trial structure. While specific student models can utilize this or not, our prediction measure treats time bins independently.

11. Only uses mc_maze_20 for real-world experiments. Need more realistic evidence, and maybe some neuroscientific insights about how few-shot decoding can better recover latent dynamics.

We added three more real world datasets, and all show the same trends.

As for insights - we don’t have groundbreaking new interpretations of the datasets from this models. But - the relation between neural activity and behavior is much more clear in the models selected by few-shot. This is despite having no access to behavior in the entire process. Furthermore, naively, the high frequency components of the latents could be noise, but could also be a true component of the data. The few-shot results strengthen the interpretation that it is noise caused by overparametrization in the student model. Figure 7(bottom) shows an example of this.

12. No statistical representation of performance comparison, but only descriptive words and figures. Need hypothesis testing to justify your assertion about data distributions (un-correlated, negatively correlated, etc).

We added p values to the correlations.

13. The authors realized using only HMMs for synthetic data is not enough, but just discussed in the appendices and provided additional results with LGSSMs. Why not integrate with the main text in the first place?

The main text now has RNNs, and briefly mentions HMMs and LGSSMs.

Minor:
- Fig 2 is a bit confusing.
- Good student on the right but mentioned first in the figure caption.
Thanks for the suggestion. We flipped good and bad students.

- What is "edge width"? Do you mean edge value or edge weight?
We clarified “Edge width” as edge thickness. The colour and the thickness both reflect the same quantity: the traffic, i.e., number of transitions when then HMM is sampled.

- There shouldn't be "invisible edges" in a graph. Maybe use lower alpha value or other colors.
We tried different variations of the graphs that include all edges and found that pruning the smallest weight edges gives the clearest picture. We included a version of the graphs with all edges visible in S6 Appendix.

- Line 201, typo, "the good student"

- L223-L225 the paragraph is just one sentence, which could be OK in some scenarios. But it looks more like the manuscript is not well organized.

- Fig 5 is not mentioned in your main text, instead you referred to Figure 19, which has duplicated contents with Fig 5.

- In your writing, you should consistently use only one of Fig , Fig. And Figure

Thank you. We corrected these in the revision.

- The real-world dataset needs a demonstration to address a non-expert audience:
- What is the experiment setting? Why should we model it with LVMs?
- How to interpret the latent variables underlying the dataset? And what does "extraneous dynamics" mean in this scenario?
- Without background information, a non-expert reader might not understand your experiments or your results, such as the "trajectories" in Fig 5 and 19, thus doubting the whole work.

We now provide a summary of four real-world datasets. Processing the neural time-series with LVMs reveals rich dynamical trajectories, at a much finer resolution than simple dimensionality reduction methods like PCA on the raw data.

Extraneous dynamics would be features of the inferred latent trajectories that are absent in the true dynamics of the neural circuit, e.g., the high-frequency ‘jitter’ in the bad model in Fig. 7 which is much less visible in the good model.

- Some important notions (smoothing, co-smoothing, few-shot co-smoothing, cross-decoding) have similar and non-intuitive names, maybe list a name table in the main text or appendix, and use abbreviations in the main text.

This is a great idea. We now include a Glossary of such terminology in the Methods section.

- Some typos ("a the" in line 89, "LGSMM" in line 478, missing section index in line 184).

Reviewer #2: This manuscript presents a new metric for evaluating latent variable models used in neuroscience, the few-shot co-smoothing score. When combined with standard co-smoothing, the new metric can identify models that fit the observed data well but that have unnecessarily complicated latent states (where the unneeded complexity is hidden by the observation model). In other words, the metric can help to identify models that have more vs less parsimonious representations of the latent dynamics. It is extremely common in modern neuroscience to fit latent state models and interpret the inferred latents in scientific terms -- so it is a huge problem that inferred latent states can be unnecessarily complicated, even when a model is a good fit to data. Hence the current manuscript is a timely and valuable contribution to the literature. It is also well-written, convincing, and thorough.

My only substantive comment is that, as the authors demonstrate, the few-shot relationship to ground truth is very sensitive to the choice of the number of few-shot neurons k, and the appropriate choice depends on the model class. So I think it is important to discuss the choice of k in the main text rather than only discussing it in the appendix.

We now discuss this in the main text, following the explanation of why few-shot works.

I include some minor additional comments below.

Minor comments:
- Equation 1: I think it would be clearer to use "Z hat" here, because f is estimating the true unknown latent state

We agree and made the changes.

- Page 5 line 118: It would be good to justify your use of xi rather than z hat, e.g. "we use its posterior probability mass function as the relevant intermediate representation because it reflects a richer representation of the knowledge about the latent state than a single discrete state estimate" or "... because it captures the degree of belief in a given latent state rather than just the most likely discrete state" or "... because the true latent state z is unknown, and xi completely summarizes the current knowledge of it"

Thank you so much. We incorporated the first option verbatim.

- Figure 4: I believe the color still represents M in this figure -- if so, please include M in the legend (like in 1D,E). Same comment for all similar figures.

Thanks, we removed the colour to simplify these figures, recognising that model size is not the focus here.
- Equations 9, 10: It wasn't obvious to me right away that xi and mu were the (posterior) probabilities of the latent states at time 1, 2. It would be good to say it explicitly.
- Equation 11: missing Bhat_1(xi) subscript
- Page 8 line 197: "we see that" -- you aren't showing the bias/variance properties here, so you should instead refer the reader to the appendix.
- Page 9 line 211: Acronym SOTA used without definition ("state of the art" is used on page 2 line 29)
- Page 9 line 236: Missing reference "as in Section we"
- Page 12 line 286: Typo "arguement"
- Page 12 line 294: Wording "may be thus can evaluated"

Thank you, we have now corrected these errors.
